# Detection of subtle neurological alterations by the Catwalk XT gait analysis system

**DOI:** 10.1186/1743-0003-11-62

**Published:** 2014-04-16

**Authors:** Ying-Ju Chen, Fu-Chou Cheng, Meei-Ling Sheu, Hong-Lin Su, Chun-Jung Chen, Jason Sheehan, Hung-Chuan Pan

**Affiliations:** 1 Stem Cell Center, Taichung Veterans General Hospital, Taichung, Taiwan; 2 Department of Education and Medical Research, Taichung Veterans General Hospital, Taichung, Taiwan; 3 Department of Neurosurgery, Taichung Veterans General Hospital, No. 160, Taichung Port Road, Sec. 3, Taichung 407, Taiwan; 4 Institute of Biomedical Sciences, National Chung-Hsing University, Taichung, Taiwan; 5 Institute of Life Sciences, National Chung-Hsing University, Taichung, Taiwan; 6 Department of Neurosurgery, University of Virginia, Charlottesville, VA, USA; 7 Faculty of Medicine, School of Medicine, National Yang-Ming University, Taipei, Taiwan

## Abstract

**Background:**

A new version of the CatWalk XT system was evaluated as a tool for detecting very subtle alteration in gait based on higher speed sample rate; the system could also demonstrate minor changes in neurological function. In this study, we evaluated the neurological outcome of sciatic nerve injury intervened by local injection of hyaluronic acid. Using the CatWalk XT system, we looked for differences between treated and untreated groups and differences within the same group as a function of time so as to assess the power of the Catwalk XT system for detecting subtle neurological change.

**Methods:**

Peripheral nerve injury was induced in 36 Sprague–Dawley rats by crushing the left sciatic nerve using a vessel clamp. The animals were randomized into one of two groups: Group I: crush injury as the control; Group II: crush injury and local application with hyaluronic acid. These animals were subjected to neurobehavior assessment, histomorphology evaluation, and electrophysiology study periodically. These data were retrieved for statistical analysis.

**Results:**

The density of neurofilament and S-100 over the distal end of crushed nerve showed significant differences either in inter-group comparison at various time points or intra-group comparison from 7 to 28 days. Neuronal structure architecture, axon counts, intensity of myelination, electrophysiology, and collagen deposition demonstrate significant differences between the two groups. There was significant difference of SFI and angle of ankle in inter- group analysis from 7 to 28 days, but there were no significant differences in SFI and angle of ankle at time points of 7 and 14 days. In the Cat Walk XT analysis, the intensity, print area, stance duration, and swing duration all showed detectable differences at 7, 14, 21, and 28 days, whereas there were no significant difference at 7 and 14 days with CatWalk 7 testing. In addition, there were no significant differences of step sequence or regularity index between the two versions.

**Conclusion:**

Hyaluronic acid augmented nerve regeneration as early as 7 days after crush injury. This subtle neurological alteration could be detected through the CatWalk XT gait analysis but not the SFI, angle of ankle, or CatWalk 7 methods.

## Background

Peripheral nerve injury has a high prevalence, and it results in a substantial societal burden from both social and economic standpoints [1,2]. Following peripheral nerve injury repair, improved behavioral outcome remains the most important evidence of functionality of axon regeneration after any repair strategy. Various behavioral testing paradigms have been developed for peripheral nerve injury research. The assessment of the functionality of axon regeneration is important in order to detect functional recovery.

The most frequently used behavioral test for evaluation of sciatic nerve injury is the walking tract analysis from which the sciatic function index (SFI) has been calculated [3]. Calculation of SFI involves measurement of paw print length and toe spreading and, therefore, requires clear prints of toe of paws. In cases hampered by flexion contractures resulting in the smearing of the print and dragging of the tail, the utility of SFI has been limited [4]. Moreover, the speed of gait, which is difficult to control using a walking track, is a confounding factor for accurately assessing various gait parameters [5]. Therefore, additional tests have been developed including gait-stance duration and kinematic ankle measurement [5,6]. Although each of these tests assesses aspects of behavioral deficits, there is still no speed-controlled behavioral test detecting a wide range of both dynamic and static gait after nerve injury.

A novel automated gait analysis system, the CatWalk 7, has the advantages of controlling speed of locomotion and automated data acquisition. It has been used in the assessment of static and dynamic gait parameters in a variety of central and peripheral nerve injury models [7-11]. Recently, we utilized CatWalk 7 system as an assessment method in peripheral nerve injury repair for either resection or crush models [12-14]. In static and dynamic determination, Catwalk 7 proved to be a reliable method to detect alteration in neurological function in keeping with cellar changes as well as the traditional neurological assessment such as SFI and ankle of angle.

The CatWalk gait analysis detects dynamic and static gait parameters. These parameters included base of support (BOS), stride length, box length, box width, maximum area, print area, mean intensity, stance duration, swing duration, regularity index (RI), and phase lags [9]. In a neuronal regeneration study, the CatWalk 7 gait analysis system demonstrated significant improvement between control and experimental groups which was consistent with improvements seen in SFI and angle of ankle testing. However, these results did not exert better detection power as compared to the tradition method. Furthermore, the sensitivity of the CatWalk 7 gait testing was not sufficient to detect the subtle alteration in the early period of nerve regeneration.

The CatWalk XT system utilizes a high speed camera with a sample rate of 100 frames per second. This sample rate is double that of Catwalk Version 7 which has a sample rate of 50 frames per second. In addition, the XT version has sharp delineation of foot prints using a combination of green light in the glass plate and red light in the illuminated ceiling. In this study, we attempt to use the CatWalk XT to detect recovery in animals undergoing peripheral nerve injury using a crushed left sciatic nerve model and then treated with hyaluronic acid, which stimulates neuronal regeneration [15].

It is not certain whether the CatWalk XT has better utility than the CatWalk 7 method for predicting the neurobehavior after peripheral nerve injury. Therefore, we conducted data comparisons between these two systems and assessed the power of detection compared to SFI, angle of ankle, electrophysiology (CMAP, conduction latency), muscle weight, and histomorphological staining such as nerve structure, axon count, axon size, and level myelination including S-100, neurofilament, Luxol fast blue and intensity of nerve fibrosis such as Sirus red. The CMAP, conduction latency, and muscle weight and histomorphology was well known for assessment of nerve regeneration, and these data were present as the basis for the comparison between these two system [3,13,16]. The nerves were retrieved at different time points for analysis of nerve regenerative markers and correlated with neurobehavioral outcomes. In particular, the neurobehavior was also correlated to axon count, axon myelination, architecture of nerve, and distribution of collagen. Based on a comprehensive analysis of nerve crush injury and subsequent nerve regenerative treatment, the Catwalk XT could provide an approach for detecting subtle functional effects resulting from neurological changes.

## Methods

### Crush models

Sprague–Dawley rats weighing 250-300 g were used in this study. The rats were anesthetized with 4% isoflurane in induction followed by a maintenance dose (1%-2%). The left sciatic nerve was exposed under a microscope using the gluteal muscle splitting method. A vessel clamp (B-3, pressure 1.5 gm/mm^2^, S&T Marketing, LTD, Switzerland) was applied 10 mm from the internal obturator canal for 20 minutes. The crush site was sutured with 9–0 nylon over the epineuria as a mark [3]. The animals were randomized into one of two groups: Group I: crush injury as the control (n = 18) with absorbable gelatin sponge soaked with 0.3 ml normal saline; and Group II: crush injury with absorbable gelatin sponge with 0.3 ml hyaluronic acid (Orthovisc) immediately after injury (n = 18). All animals received rehabilitation therapy on a metal mesh every week. Food and water were provided ad libitum before and after the experiments. The animals were kept in a temperature-controlled environment at 20°C, and were exposed to alternating light and dark cycles of 12 h. All animals were treated and cared for in accordance with the guidelines recommended by the Ethics Committee of Taichung Veterans General Hospital.

### Analysis of functional recovery

A technician who was blinded to treatment allocation evaluated the sciatic nerve function weekly after the surgery. The evaluation method included ankle kinematics and sciatic function index (SFI) [14,17]. In the sagittal plane analysis, the ankle angle was defined by the intersection of line extending from the knee to ankle joint and from the ankle joint to metatarsal head. The angle was expressed as the degree at terminal stance (i.e. the last moment at which the foot was in contact with the ground).

Several measurements were taken from the footprint by red ink print, and these included the following: (i) distance from the heel to the third toe, the print length (PL); (ii) distance from the first to fifth toe, the toe spread (TS); and (iii) distance from the second to the fourth toe, the intermediary toe spread (ITS). All three measurements were taken from the experimental (E) and normal (N) sides. The SFI was calculated according to the equation:

$$\begin{array}{l} \mathrm{SFI}=-38.3\left(\mathrm{EPL}-\mathrm{NPL}/\mathrm{NPL}\right)+109.5\left(\mathrm{ETS}-\mathrm{NTS}/\mathrm{NTS}\right)\\ \;+13.3\left(\mathrm{EIT}-\mathrm{NIT}/\mathrm{NIT}\right)-8.8. \end{array}$$

The SFI oscillates around 0 for normal nerve function, whereas an SFI of approximately −100 represents total dysfunction.

### CatWalk-automated quantitative gait analysis

The core of the CatWalk system consists of an enclosed walkway on a glass plate that is traversed by a rodent from one side of the walkway to the other. Green light enters at the long edge of the plate and is completely internally reflected [7]. Light is able to escape only at the areas where the animal’s paw make contact with the glass plate, and, as a result, the light is scattered. Using Illuminated Footprint™ technology, paws are captured by a high speed video camera that is positioned underneath the walkway. The CatWalk XT system includes a high speed digital camera with a sample rate of 100 frames per second, and the frame rate of the camera used in CatWalk 7 was 50x per second. The brightness of a pixel depends on the amount of light received from such an area by the camera. The Illuminated Footprint™ enables intensity difference to be detected between animals’ paws.

The 3D footprint intensity tab plots the print intensity of the 4 paws for each individual frame in which the paws have contact with the glass plate in a 3D chart. The intensities vary from 0 to 225 and are represented by different colors. A 3D chart can be rotated in all directions.

The noteworthy differences between CatWalk 7 and XT include setting, acquisition, analysis, and camera system. The details of difference are detailed in Table 1. In brief, the setting in CatWalk 7 involved multiple setting with no quantitative feedback of the outcome. However, in CatWalk XT, there were fewer settings with quantitative feedback of the outcome. CatWalk XT offers a function that searches for the optimal setting automatically. In CatWalk 7, footprints are captured at a speed of 50 Hz (PAL) to 60Hz (NTSC). In CatWalk XT, footprints are captured at a speed of 100Hz high with a high speed color camera. Regarding specific properties of the camera system, the CatWalk 7 uses a TM-62EX (Pulnix, USA) camera positioned perpendicularly to the center of the Catwalk glass plate. The camera lens’ physical attributes are 8.5 mm size and 62.4 degree curvature (Cosmica, Japan). However, the CatWalk XT uses a GP-3360 camera (Gevicam, USA) positioned perpendicularly to the center of Catwalk glass plate. The CatWalk XT system’s lens is 8.5 mm in size and 65 degree curvature (Fujinon, China). The video camera transforms each scene (i.e. the area in front of the lens) into a digital image (i.e. an image composed of discrete pixels of digital brightness values). The digital images are transferred to a computer through an Ethernet connection. During the analysis as part of the CatWalk 7 system, footprints visible on the images are manually classified. However, in the CatWalk XT, footprints are automatically classified by the system. Other body parts that contact the glass plate are highlighted so that they can be manually labeled or discarded. The CatWalk XT also has new functions of automatic footprint classification, error correction, interactive footprint measurements, and data segmentation profiling.

**Table 1:** **Different operation system between CatWalk 7 and XT (summaried according to website:** **http://www.noldus.com/ )**

**Version**	**CatWalk 7**	**CatWalk XT**
Settings	I. Detection settings involve multiple settings with no quantitative feedback of the outcome.	I. Detection settings involve fewer settings with quantitative feedback of the outcome. CatWalk XT even offers a function that searches for the most optimal settings automatically.
	II. Recording of video is based on defining start and stop zones, which can be swapped depending on the direction of movement of the animal in the walkway.	II. Recording of video is performed automatically.
	III. Track files are given unique descriptions based on animal ID, run ID, and time.	III. User can define a trial list that includes animal ID, experiment group, and time point. These independent variables can later be used for data selection.
Acquisition	I. Footprints are captured at a speed of 50 Hz (PAL) to 60 Hz (NTSC).	I. Footprints are captured at a speed of 100Hz high with a high speed color camera.
	II. Acquisition must take place in complete darkness.	II. Acquisition can take place under red ambient light conditions.
	III. Runs are captured if a user-definable maximum duration has not been reached.	III. Runs are labeled “compliant” if the run complies with user-definable duration interval and speed variation. All runs are captured, but for analysis, one can choose to only select “compliant” runs.
	PAL: Phase Alternating Line	IV. Feedback is provided during acquisition when a user-definable number of “compliant” runs have been captured.
	NTSC: National Television System Committee	
Analysis	I. Footprints are manually classified based on a black image where only footprints are visible.	I. With Automatic Footprint Classification, footprints are automatically classified. Other body parts that contact the glass plate are highlighted, so that these can easily be manually labeled or discarded.
	II. CatWalk provides a list of parameters, for a detailed assessment of the locomotion of your animal.	II. Video export for presentation purposes.
		III. CatWalk XT provides data on three levels of abstraction:
		➢ raw data on video frame-by-frame basis
		➢ run statistics (equivalent to software 7.1)
		➢ trial statistics which provide the average for multiple runs that belong to the same trial (per animal, per time point) as if it was one combined run.
Camera	Lens: 8.5 mm, Cosmicar, Japan Camera: TM-62EX, Pulnix, USA	Lens: 8.5 mm, Fujinon, China Camera: GP-3360, Gevicam, USA

Quantitative analysis of the data from the Catwalk XT included the following parameters as previous described [7]:

1. Step sequence distribution--Six different walking patterns or normal step sequence patterns can be discerned in rats, depending on the sequential placement of the four paws, which fall into three different categories (i.e. cruciate, alternate, rotary).

2. Regularity (RI)--This parameter is a measure of inter-limb coordination. Inter-limb coordination is considered normal when during uninterrupted locomotion only regular step sequences are used. The regularity index rates the degree of inter-limb coordination as a percentage of complete coordination by the following equation:

$$\mathrm{RI}=\left(\mathrm{NSSP}\times 4/\mathrm{PP}\right)\times 100\%,$$

where NSSP represents the number of normal step sequence patterns and PP the total number of paws placements. Consequently, extra paw placements and irregular walking on three paws would result in a decrease in the RI.

3. Print area--This parameter is defined as the total floor area in pixels contacted by the paw during stance phase. Possible reasons for an increase in the hind limb print area are paralysis of the lower limb leading to a deficiency in plantar stepping or paw/toe dragging during part of the step cycle. A decrease in this parameter can be indicative of mechanical allodynia [9].

4. Base of support--The distance in millimeters (mm) between the two hind paws is defined as the base of support. This distance is measured perpendicularly to the direction of walking.

5. Duration of the swing and stance phases--Since the duration of the stance or swing phase depends on the animal’s walking speed and degree of dysfunction, these parameters are transformed to a fraction of the total step duration according to the following formula: fraction stance or swing phase = [time in stance or swing phase/(time in single step)] × 100%. Durations of the swing or stance phase and total step are expressed in seconds.

6. Hind paw pressures--This is the mean intensity of the contact area of the hind paw at the moment of maximal paw-floor contact. This parameter is expressed in arbitrary units (a.u.).

### Electrophysiologic assessment

Sciatic nerves from individual groups were exposed 4 weeks after operation, and electrical stimulation was applied to the proximal side of the injured site. The evoked compound muscle action potential (CMAP) amplitudes and conduction latencies were recorded in the gastrocnemius muscle with an active monopolar needle electrode 10 mm below the tibia tubercle and with a reference needle 20 mm from the active electrode. The stimulation intensity and filtration ranges were 5 mA and 20–2000 Hz, respectively. A similar assessment was performed on the non-injured side. The CMAP data and conduction latency were converted to a ratio of the injured side divided by the normal side so as to adjust for the effect of anesthesia [3].

### Immunohistochemistry

Serial 8 μm-thick sections of sciatic nerve were cut using a cryostat, mounted on superfrost/plus slides (Menzel-Glaser, Braunschweig, Germany) and subjected to immunohistochemistry with antibodies against S-100 and neurofilament for detection of nerve regeneration at the intervals of 7 days. The immunoreactive signals were observed using goat anti-mouse IgG (FITC) (Jackson, 1:200 dilution), anti-mouse IgG (Rhodamine) (Jackson, 1:200 dilution), or 3, 3’-diaminobenzidine brown color. For determining the objects of interest, six nerves in each group were cut longitudinally into 8 μm-thick sections and stained with each antibody. The region of maximum diameter of the resected nerve tissue with crush mark was chosen for examination. Areas of activities (0.2 mm^2^) appeared dense against background and were measured by a computer image analysis system (Alpha Innotech Corporation, IS 1000).

### Histological examination

After neurobehavioral and electrophysiological testing, six rats in each group underwent transcardial perfusion with 4% paraformaldehyde in 0.1 M phosphate buffer (pH 7.4) after being re-anesthetized. The left sciatic nerve was harvested from the animals after electrophysiological testing, and the nerve tissue was fixed on a plastic plate using stay-suturing to keep the nerve straight. Bilateral gastrocnemius muscle from the bones was sent for measurement of muscle weight.

The obtained nerve tissues (5 mm) distal to the crush (5 mm) were fixed in 3% glutaraldehyde solution, post-fixed in 0.5% osmium tetroxide, embedded, cut transversely into 4 μm thick sections and stained with toluidine blue 4 weeks after operation. The number of axons was counted in 10 randomly selected fields (0.1 mm^2^) at a magnification of 200 × .

Six nerves in each group were embedded, cut longitudinally into Sections 8 μm thick, and stained with haematoxylin-eosin (H&E) for the measurement of vacuole number and Luxol fast blue and Sirus red for determination of collagen deposition. The determination of numbers of vacuoles and density of Luxol fast blue and Sirus red have been previously described in detail [16].

### Statistical analysis

Data are expressed as mean ± standard error (SE). The statistical significance of differences between groups was determined by one–way analysis of variance (ANOVA) followed by Dunnett’s test. For SFI and Catwalk analysis, the results were analyzed by repeated-measurement ANOVA followed by Bonferroni’s multiple comparison method. A *p* value less than 0.05 was deemed statistically significant.

## Results

### Hyaluronic acid increased neuronal regeneration and improved neurobehavior

**Figure 1:**
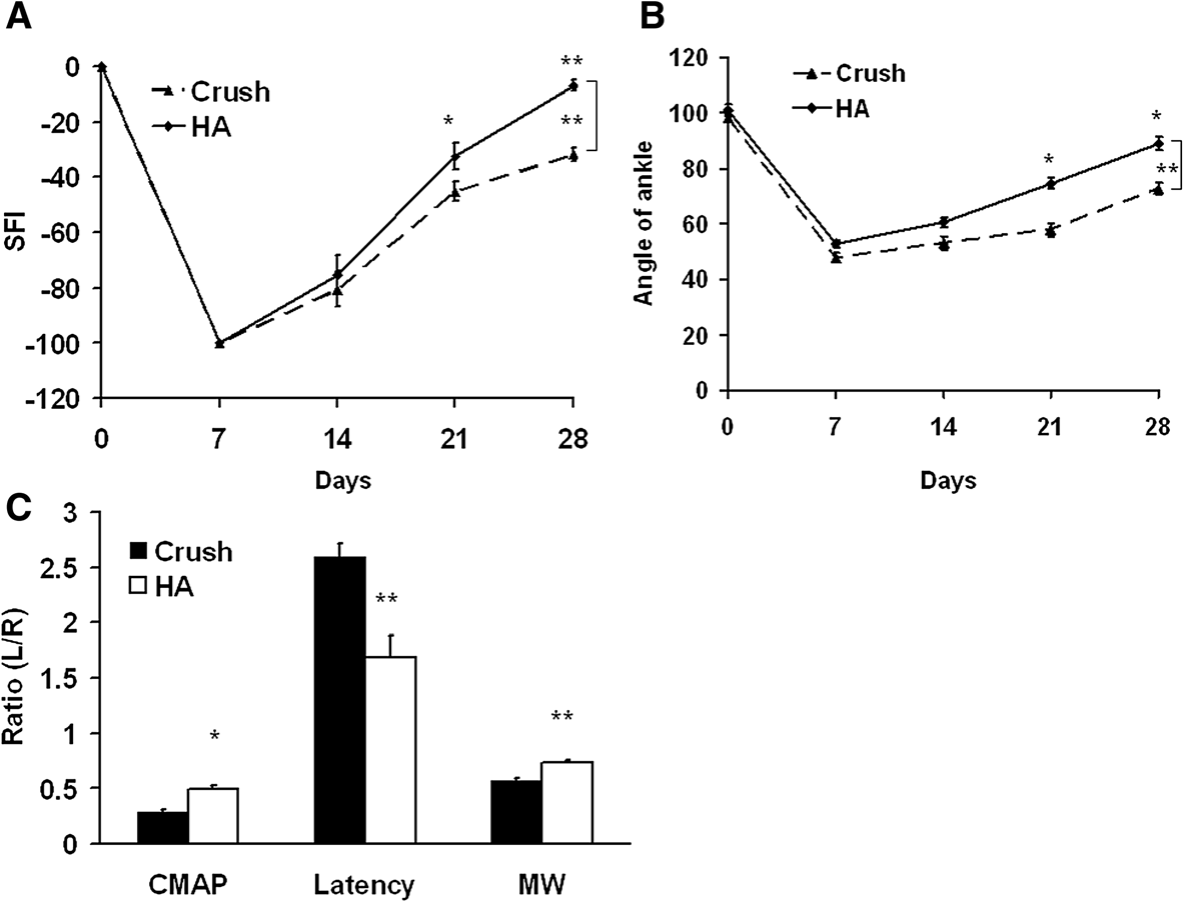
**Evaluation of SFI, angle of ankle, electrophysiological function, and muscle weight.** Before crushing injury, these animals received basal line assessment including SFI and angle of ankle. These animals were subjected to left sciatic nerve crush injury and allowed to receive examination of SFI and angle of ankle at time points of 7, 14, 21, and 28 days. At the time point of 28 days, the animals were allowed to receive electrophysiological evaluation and muscle weight. **(A, B)** Representative scores of SFI and angle of ankle are depicted with respect to different time profiles. **(C)** The graph details the results of CMAP, conduction latency, and muscle weight. Crush; HA groups: see text. *p < 0.05; **p < 0.01; n = 6.

Increased neuronal regeneration was accompanied by an improvement in sciatic nerve function index, increased compound muscle action potential, reduced nerve conduction latency, and increased muscle weight. The values of SFI and angle of ankle are shown in Table 2. Treatment with hyaluronic acid (HA) exerted a significant improvement on SFI compared with findings obtained with crush injury alone (p < 0.001, F = 19) (Figure 1A) in the intergroup analysis. The ankle of angles between the HA treated animals and untreated controls also showed the same trend (p < 0.001, F = 25) (Figure 1B). However, there were no significant differences in SFI and angle of ankle at 7 and 14 days, respectively.

**Table 2:** Representative of SFI and angle of ankle in different treatment group related to different time frame

**Parameter**		**Days**				
	**Group**	**0**	**7**	**14**	**21**	**28**
SFI	Crush	0 ± 0	−100 ± 0	−80.5 ± 6.4	−45.1 ± 3.5	−31.8 ± 2.3
	HA	0 ± 0	−100 ± 0	−75.3 ± 7.1	−32.56 ± .8	−6.9 ± 1.9
Angle of ankle	Crush	98.2 ± 2.2	47.7 ± 1.1	53.2 ± 1.7	57.8 ± 1.9	72.8 ± 2.4
	HA	101 ± 1.3	52.8 ± 1.6	60.5 ± 2.4	74.6 ± 2.3	89.1 ± 2.1

The data was present as mean ± standard error.

Crush, HA, angle of ankle and SFI: see text.

**Figure 2:**
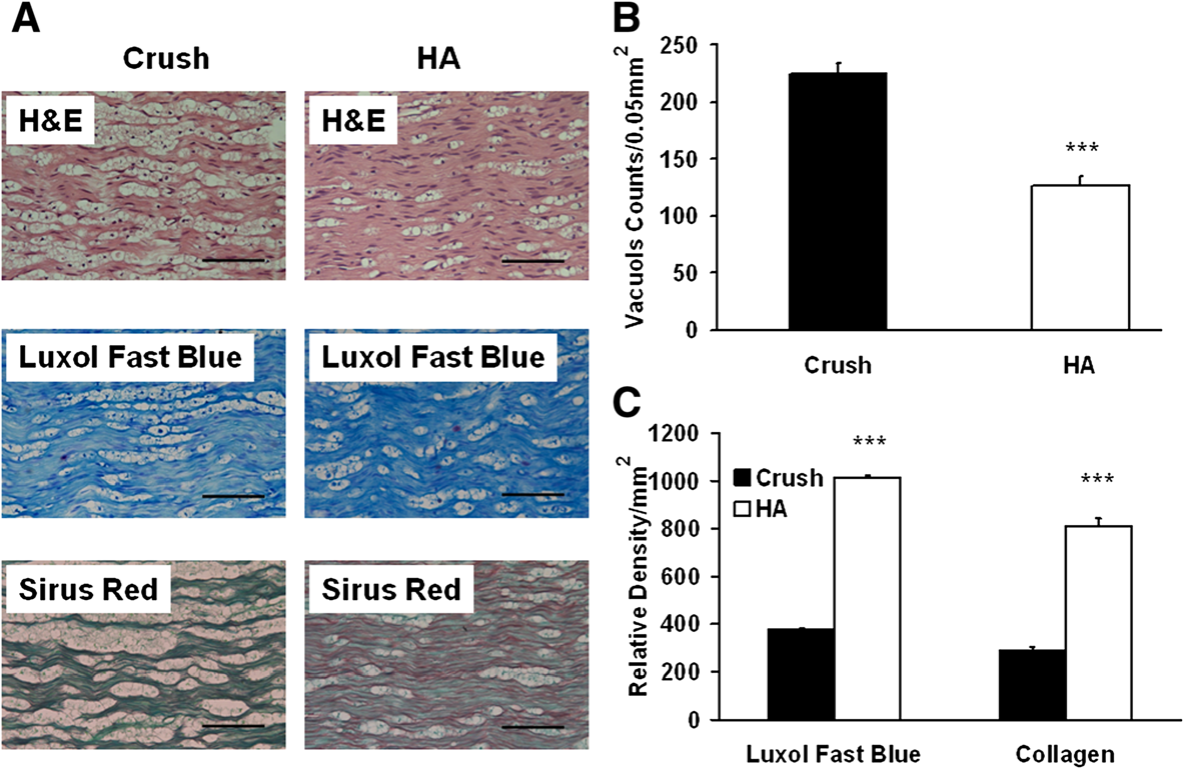
**Vacuole counts, Luxol fast blue, and Sirus red staining 4 weeks after hyaluronic acid treatment.** After neurobehavioral assessment and electrophysiological examination, the injured nerve were harvested for assessments **(A)** Representative photomicrographs illustrating vacuole, Luxol fast blue, and Sirus red staining in each group, **(B)** Quantitative analyses of vacuole counts, **(C)** Quantitative analysis of, Luxol fast blue, and collagen density. ***p < 0.001; Bar length = 50 μm, n = 6.

Following a similar trend of improvement, the parameters of CAMP, conduction latency, and muscle weight also were restored by HA from 28 ± 3% to 49 ± 4% (p < 0.001), 260 ± 12% to169.2 ± 19% (p < 0.001), and 57 ± 3% to 73 ± 2% (p < 0.001), respectively (Figures 1C) at 28 days following nerve injury. Increased myelination and vascular organization as well as decreased number of vacuoles were positively correlated to the integrity of nerve tissue, and these findings reflected the degree of nerve regeneration in the later phase. The significant improvement in the parameters of nerve regeneration such as vacuole number (from 224 ± 9.7 to 126.8 ± 8.3 counts/0.05 mm^2^; p < 0.001) and myelination as evidenced by Luxol fast blue (from 377 ± 15.9 to 1013.7 ± 32.9 relative density/mm^2^; p < 0.001) were demonstrated in the HA group at 28 days following injury. Furthermore, the significant expression of collagen was shown in HA (from 288.2 ± 11.3 to 810.5 ± 31.1 relative density/mm^2^; p < 0.001) (Figure 2) at 28 days following injury.

**Figure 3:**
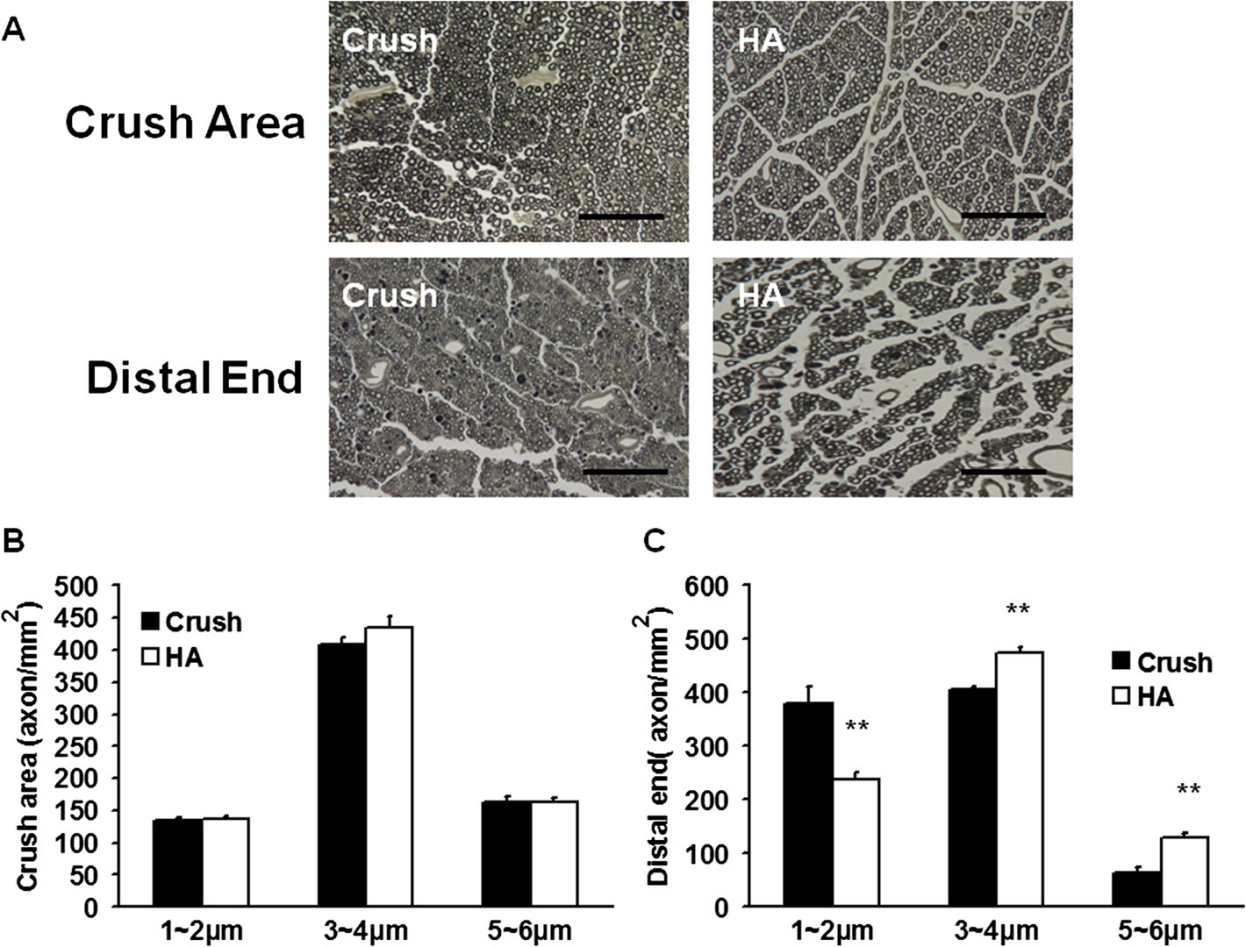
**Illustration of size and number of axon 4 weeks after hyaluronic acid treatment.** The injured nerve was retrieved for assessment axon size and counts over the injured area and distal end of crushed nerve. **(A)** Representative photomicrography of morphology and number of axon in the crush and distal end of crush nerve **(B)** Quantitative analysis of various sizes of axon in the crush area **(C)** Quantitative analysis of various sizes of axon in distal end of the crushed nerve. **p < 0.01; Bar length = 50 μm, n = 6.

Increased axonal size was demonstrated in the HA group, and this finding was consistent with a significant improvement in neurological function. The distribution of axon size and location of sampling is shown in Table 3. In this study, hyaluronic acid treatment exerted a significant increase in larger size axons (5-6 um), but HA treatment also led to an accompanying decrease in smaller size axon (<2 μm) over the distal end of crushed nerve at 28 days after nerve injury (Figure 3A, C). However, there was no significant change over the crushed site either with or without HA treatment (Figure 3A, B). Based on this portion of the study, HA treatment significantly improved the nerve regeneration of crush injured animals as compared to untreated control animals.

**Table 3:** Representative of axon size in different treatment and location

**Location**		**Size**		
	**Treatment**	**1-2 μm**	**3-4 μm**	**5-6 μm**
Distal end of nerve	Crush	134 ± 5.6	408 ± 11.5	163 ± 9.2
	HA	136 ± 4.7	435 ± 15.3	162 ± 7.8
Crushed site of nerve	Crush	381 ± 31.1	405 ± 12.2	64 ± 6.2
	HA	237 ± 14.5	474 ± 11.3	130 ± 8.9

The data was present as number of axon counts/mm^2^.

### Assessment of nerve regeneration by S-100 and neurofilament in inter-and intra-group comparison

**Figure 4:**
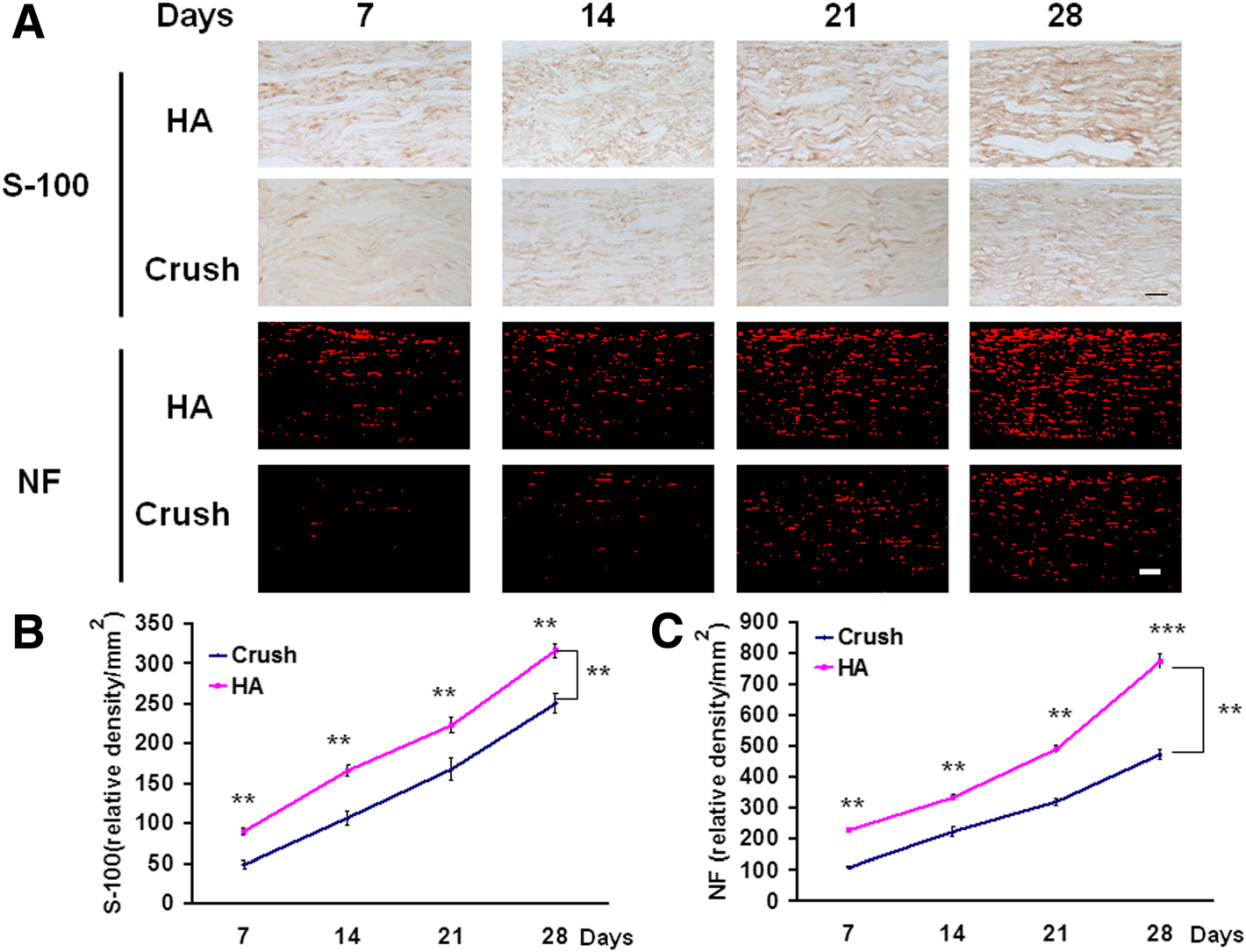
**Expression of nerve regeneration plotted against with time course.** The injured nerves were harvested at various time points of 7, 14, 21, and 28 days after nerve crush. These nerves were subjected to immunohistochemistry staining of S-100 and neurofilament. **(A)** Microphotograph showed the expression of S-100 and NF in different treatment related to various time points. **(B)** Quantitative analysis of S-100 at different time points **(C)** Quantitative analysis of NF at different time points. **p < 0.01; ***p < 0.001, Bar length = 100 μm; n = 3. HA and NF: see text.

For comprehensive investigation of neuronal regeneration, the early (neurofilament) and mature (S-100) nerve regeneration makers were used to assess the effect of HA treatment over time. The distribution of S-100 and neurofilament as a function of treatment and time frame are shown in Table 4. HA treatment exerted a significantly increased expression of S-100 and neurofilament in the distal end of crushed nerve (Figure 4A) at inter-group comparison at time points of 7, 14, 21, and 28 days (Figure 4B, C). Either in crush or HA group, the intra-group differences were also present at the time points of 7, 14, 21, and 28 days (Figure 4B, C). These observations demonstrate that consecutive nerve sampling through measuring S-100 and NF is an appropriate analytical method to evaluate for intra- and inter-group differences.

**Table 4:** The distribution of S-100 and neurofilament related to different treatment at different time frame

**Parameter**		**Days**			
	**Group**	**7**	**14**	**21**	**28**
S-100	Crush	48.3 ± 4.4	107 ± 6.5	168 ± 10.1	250 ± 8.7
	HA	90 ± 5.7	166 ± 8.8	223 ± 14.5	316 ± 12.1
NF	Crush	108 ± 6.1	223 ± 14.5	320 ± 11.5	473 ± 14.5
	HA	228 ± 7.3	333 ± 8.8	490 ± 12.2	773 ± 23.3

S-100 and NF: data was present as mean ± standard errors with relative density/mm^2^.

S-100 and NF: see the text.

### Subtle neurological dysfunction detected by the CatWalk system

**Figure 5:**
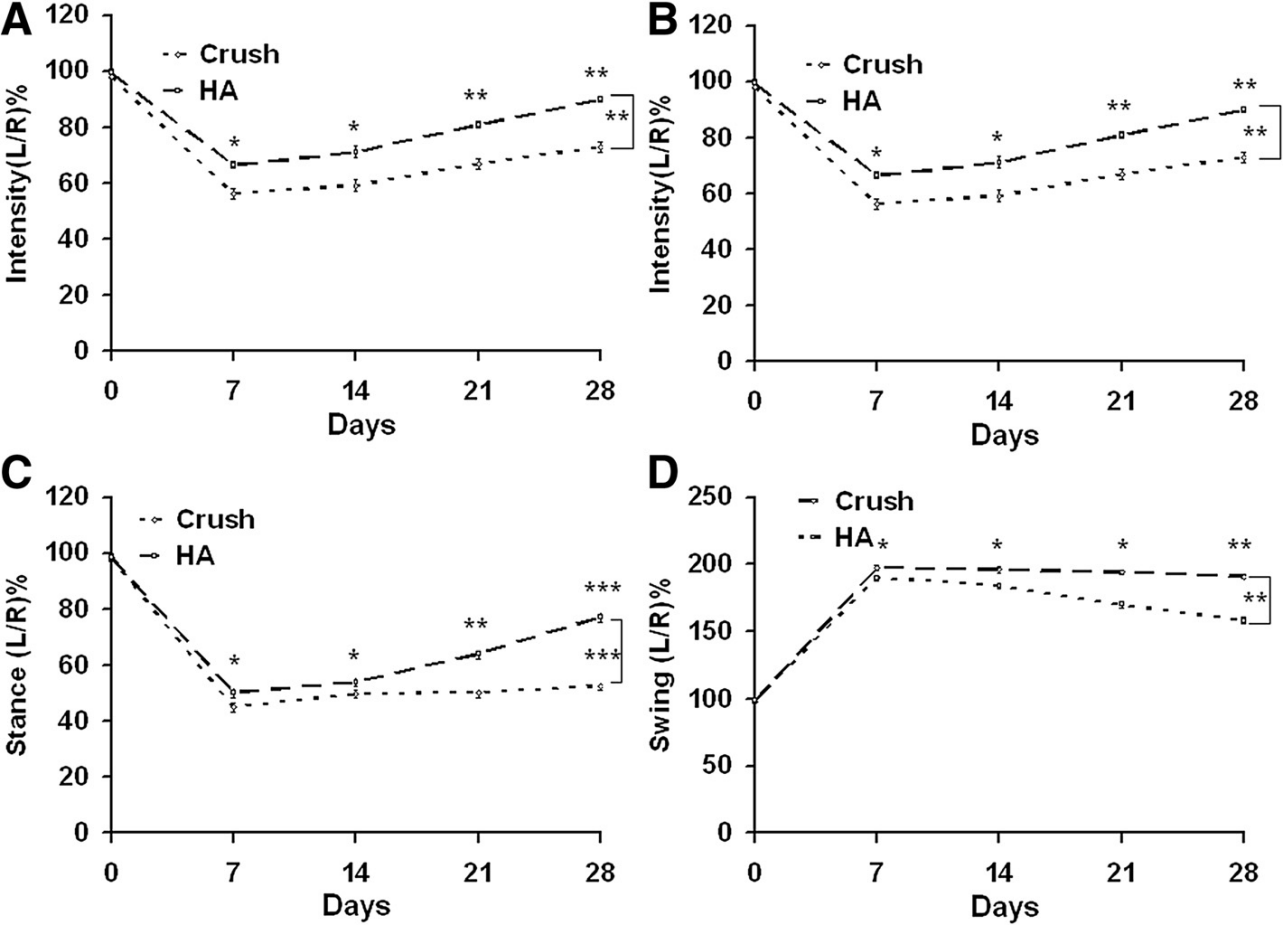
**Representative of CatWalk XT analysis related to different time points. (A)** Ratio of mean intensity (L/R), **(B)** Printed area ratio (L/R), **(C)** Stance duration ratio (L/R), and **(D)** Swing duration ratio (L/R). *p < 0.05; **p < 0.01; ***p < 0.001, n = 6, L: left, R: right.

In the Catwalk analysis with version XT (Table 5), the mean intensity and printed area showed significant improvement in the HA group compared to the crush groups (p < 0.01, F = 7.1; p < 0.01 F = 8.1) (Figures 5A, B). In stance duration, the ratio of duration dropped to half of the original value and then exhibited further improvement in the HA group (p < 0.001, F = 21.5) (Figure 5C). In the swing duration, the ratio of duration showed a significant decrease in HA group compared to the crush group (p < 0.01, F = 8.2) (Figure 5D). However, there was no significant difference in step sequence or regularity index between the two groups (data not shown). At various time points between 7 to 28 days after injury, there were significant differences between the crush and HA treated groups in mean intensity (p < 0.05, p < 0.05, p < 0.01, p < 0.01), printed area (p < 0.05, p < 0.05, p < 0.01, p < 0.01), swing duration( p < 0.05, p < 0.05, p < 0.01, p < 0.001), and stance duration (p < 0.05, p < 0.05, p < 0.05, p < 0.01). The significant differences at each time point were consistent with the results in S-100 and neurofilament expression. Furthermore, the intensity, printed area, swing duration, and stance duration results derived from the Catwalk XT system proved more sensitive in detecting neurological differences between groups than the SFI and angle of ankle.

**Table 5:** Representative of CatWalk XT parameter related to different time frame

**Time (days)**		**Parameter**			
	**Treatment**	**Intensity**	**Print area**	**Stance duration**	**Swing duration**
0	Crush	98.1 ± 2.1	98.2 ± 1.83	98.3 ± 1.2	98.3 ± 1.1
	HA	99.2 ± 1.7	98.8 ± 1.6	98.5 ± 1.3	98.5 ± 1.2
1	Crush	57.3 ± 1.9	24.2 ± 0.8	45.9 ± 1.8	198.5 ± 1.3
	HA	56.8 ± 1.3	23.7 ± 0.7	46.1 ± 1.7	198.2 ± 1.7
7	Crush	56.3 ± 2.1	16.1 ± 1.1	47.8 ± 1.8	197.5 ± 1.8
	HA	66.5 ± 1.2	23.1 ± 0.9	50.3 ± 1.8	190.2 ± 1.9
14	Crush	59.2 ± 2.3	20.1 ± 1.3	49.8 ± 1.7	196 ± 2.3
	HA	71.2 ± 2.2	31.1 ± 1.6	54.1 ± 1.4	184.1 ± 2.1
21	Crush	67.1 ± 1.9	27.2 ± 1.3	50.3 ± 1.6	194 ± 1.9
	HA	81.1 ± 1.5	40.1 ± 1.4	64.1 ± 1.7	170.3 ± 2.2
28	Crush	73.1 ± 1.7	35.3 ± 1.1	52.6 ± 1.8	191 ± 1.7
	HA	90.2 ± 2.2	55.2 ± 1.3	77.1 ± 1.5	158.1 ± 2.1

**Figure 6:**
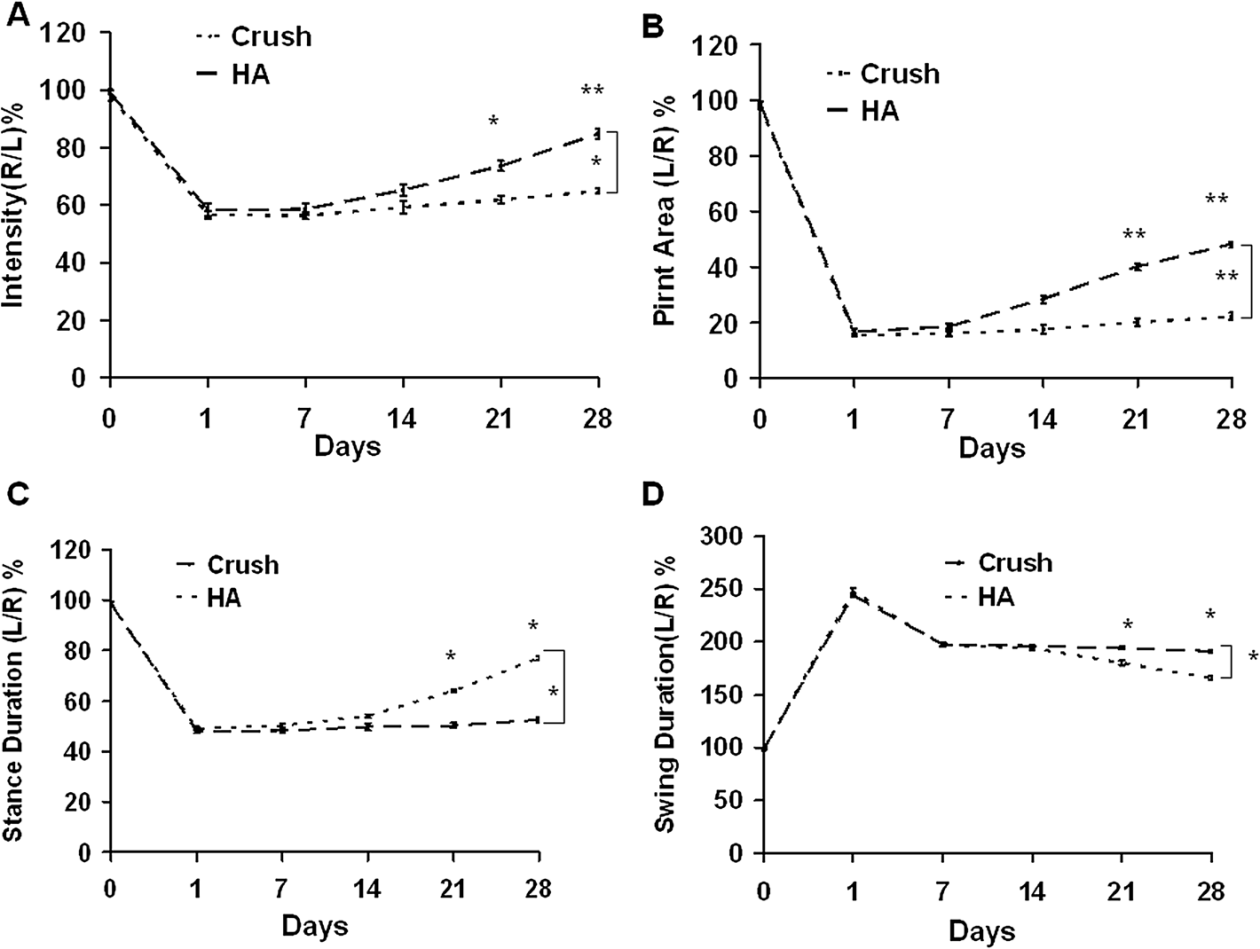
**Representative of CatWalk 7 gait analysis related to different time points. (A)** Ratio of mean intensity( L/R), **(B)** Printed area ratio (L/R), **(C)** Stance duration ratio (L/R), and **(D)** Swing duration ratio (L/R). *p < 0.05; **p < 0.01; n = 6, L: left, R: right.

In analysis of Catwalk version 7 (Table 6), the mean intensity and printed area showed significant improvement in the HA group compared to crush groups (p < 0.05; p < 0.01) (Figures 6A, B). In stance duration, the ratio of duration dropped to half of the original value and then exhibited further improvement in the HA group (p < 0.05) (Figure 6C). In the swing duration, the ratio of duration showed a significant decrease in the HA group compared to the crush group (p < 0.05) (Figure 6D). However, there was no significant difference in step sequence or regularity index between the two groups (data not shown). At various time points between 14 to 28 days, there were significant differences between crush and HA groups in mean intensity (p < 0.05, p < 0.01), printed area (p < 0.01, p < 0.01), swing duration (p < 0.05, p < 0.05), and stance duration (p < 0.05), but there were no significant difference at the 7 and 14 day time points. However, the results showed that the XT version afforded a better detection rate than that version 7, and XT’s detection rate was also superior to SFI and angle of ankle metrics alone.

**Table 6:** Representative of CatWalk 7 parameters related to different time frame

**Time (days)**		**Parameter**			
	**Treatment**	**Intensity**	**Print area**	**Stance duration**	**Swing duration**
0	Crush	98.1 ± 1.8	98.2 ± 1.7	98.3 ± 1.5	98.3 ± 1.7
	HA	99.2 ± 1.7	98.8 ± 1.6	98.5 ± 1.2	98.5 ± 1.2
1	Crush	56.5 ± 2.2	15.6 ± 1.1	48.1 ± 0.9	198.9 ± 1.7
	HA	55.9 ± 1.5	15.9 ± 1.2	48.3 ± 1.5	197.9 ± 2.8
7	Crush	56.3 ± 2.1	16.1 ± 1.2	48.3 ± 1.2	197.5 ± 1.1
	HA	58.5 ± 1.2	18.6 ± 0.9	50.3 ± 1.1	197.2 ± 1.9
14	Crush	59.2 ± 2.3	1.76 ± 1.1	49.8 ± 1.7	196.1 ± 1.3
	HA	65.2 ± 2.2	28.6 ± 1.6	54.1 ± 1.3	194.3 ± 1.8
21	Crush	61.8 ± 1.8	20.1 ± 1.2	50.3 ± 1.5	194.2 ± 2.1
	HA	73.6 ± 1.3	40.1 ± 1.4	64.1 ± 1.1	180.1 ± 2.3
28	Crush	64.8 ± 1.7	22.3 ± 1.1	52.6 ± 1.6	191.2 ± 2.9
	HA	84.8 ± 1.8	48.2 ± 1.3	77.1 ± 1.7	166.1 ± 2.1

**Figure 7:**
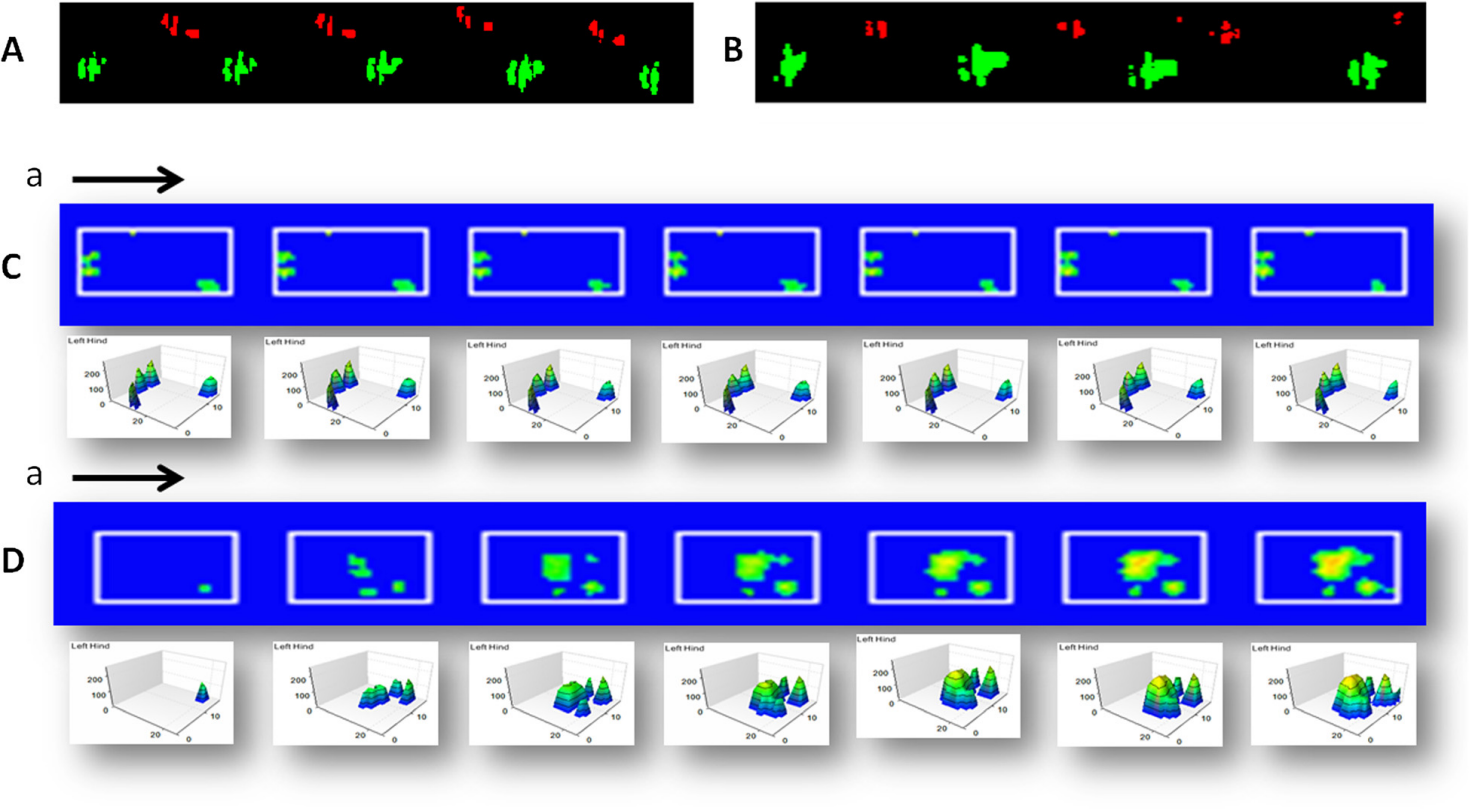
**Representative foot prints as detected by the CatWalk 7 and CatWalk XT system.** Left sciatic nerve crushed animals treated either with or without HA was allowed to receive CatWalk 7 and CatWalk XT gait analysis at time points of 7 days after injury. **(A)** Illustration of foot prints using the CatWalk 7 analysis in the crush group. Red showed the foot prints of the left foot (crushed injury) and green showed the foot prints of the right leg (normal side). **(B)** Illustration of the foot prints using CatWalk 7 analysis in the HA group. Red showed the foot prints of left foot (crushed injury) and green showed the foot prints of the right leg (normal side). **(C)** Illustration of consecutive seven foot prints in a single step from toe on using the CatWalk XT system for the crush group. The steps sequence was from left to right. The upper row of blue boxes depicted 2 D gait and each figure below the respective blue box indicated the 3 D gait. **(D)** Illustration of a consecutive set of seven foot prints in a single step from toe on using the CatWalk XT system for the HA group. The steps sequence was from left to right. The upper row of blue boxes showed 2 D gait and each figure below the blue box indicated the 3 D gait analysis. The color was representative of increased density from green to yellow. Green color demonstrated low density and yellow color depicted high density. The data of intensity was the summation of all intensity from all boxes from the toe on. a: indicated the toe on of the first step. arrow: indication of direction of the steps. HA: see text.

Furthermore, in the XT version of the CatWalk gait analysis, two dimensions of foot prints at a limited time frame were reconstructed to three dimensions of foot prints through a summation of density, which showed the dynamic change from the toe on to toe off. At 7 days post injury, there was no significant difference detected by CatWalk 7 system (Figure 7A, B). However, there were significant differences in summation intensity between crush (72 ± 4.5 a.u.) and HA (159 ± 7.8) (p < 0.001) groups using the CatWalk XT system. The XT system detecting of subtle gait differences is illustrated in Figure 7C and D which depicts seven steps from the toe on. These findings were consistent with trends in nerve regeneration markers of S-100 and neurofilament at 7 days. These results further demonstrated that the XT version of the CatWalk gait analysis revealed subtle neurological dysfunction which could not be determined by cruder measures such as SFI and angle of ankle or the earlier CatWalk 7 analytical program.

## Discussion

CatWalk XT gait analysis detailed the foot print using a high speed sampling rate and provided better spatial and imaging resolution. The results from this study showed a significant improvement in gait analysis especially in subtle neurological dysfunction. This subtle neurological alteration could be determined using the XT system as early as 7 days following crush injury, and the same alteration was not detected in the version 7 Catwalk system nor was it detected as well using the tradition methods of analyzing SFI and angle of ankle alone. There were two arms to facilitate high detection power by the intra-group comparison as well as inter-group comparison by intervening with the application of hyaluronic acid. Nerve regeneration was determined by the restoration of architecture of nerve, increased axon number, increased myelination, deposits of collagens. Through inter- and intra- groups’ comparison, the CatWalk XT system demonstrated better accuracy than the CatWalk version 7 system.

Hyaluronic acid is an agent which is known to reduce the extent of scar formation by inhibiting lymphocytes migration, proliferation and chemotaxis, granulocyte phagocytosis and degranulation, and macrophage motility [18]. Topical application of HA can facilitate nerve regeneration process by preventing epineural and extraneural scar formation [15]. In this study, local application of HA not only increased nerve regeneration in neurobehavior, increased axon myelination, restoration of nerve architecture but also the increased collagen distribution. Based on these findings, HA intervention caused subtle neurological improvement as early as 7 days. This early neurological improvement could be determined by CatWalk XT gait analysis, and the improvements in gait detected by the CatWalk XT gait analysis were in line with consecutive nerve sampling for S-100 and neurofilament.

During nerve regeneration, myelination of nerve fibers was used as a hallmark to determine the intensity of nerve regeneration [19]. Neurofilament expression indicated the early evidence of nerve regeneration potential [20]. The amount of S-100 immunoreactivity in myelinated fibers appeared to be directly correlated with thickness of myelin sheath formed by Schwann cells [21]. Hence, in this study, we retrieved injured nerve to determine the immunoreactivity of neurofilament and S-100 at different time points, and we correlated this immunoreactivity expression with the data from the CatWalk XT gait analysis. These results revealed subtle alterations in the CatWalk XT gait analysis consistent with the expression of immunoreactivity of neurofilament and S-100. This correlation further confirmed our hypothesis that CatWalk XT gait analysis can be a research instrument and provide better accuracy than the CatWalk 7 system.

Spontaneous recovery of motor function in a sciatic nerve crush injury model has been discussed several decades ago, and it has recently become a topic of renewed interest [22,23]. It should be pointed out that in this study the potential for spontaneous recovery exists even without any treatment. Without executing a serious nerve damage model such as nerve transection, the detection of nerve regeneration by the CatWalk gait analysis should be interpreted carefully. Based on our previous studies, we found that nerve regeneration in a crush experimental model did not reach full recovery and left serious neurological deficits at the 4 week time point [3,13]. Using a cut off value of 4 weeks for evaluating neurological deficits, electrophysiological parameters, or even more importantly in nerve myelination, there were significant differences between groups, which could be used as a valuable test to assess the power of CatWalk system in nerve regeneration.

The CatWalk XT system comes with a high speed digital camera. The digital camera has a sample rate of 100 frames per second. The frame rate of the camera used in CatWalk 7 (old versions) was 50 (PAL) or 60 (NTSC) fields per second, so the new frame rate doubles the temporal and image resolution. In addition, the combination of green light in the glass plate and red light in the illuminated ceiling makes the body contour of an animal visible when it crosses the glass plate, and this increases paw print classification. In this study, we found that there were no significant differences of neurobehavior as early as 7 days after injury based on SFI and angle of ankle as well as the CatWalk version 7. In the CatWalk XT, it provides the improvement in hardware and facilitated the detection of early and subtle neurological changes in the crush peripheral nerve model.

For multimodal gait analysis, it is usually recommended to combine behavioral testing (SFI, angle of ankle, CatWalk) with other test like electrophysiological tests (nerve conduction velocity, compound muscle action potential) and histomorphometrical test (e.g. myelin thickness, axon diameter, expression of molecular marker) [12,13]. The CatWalk system has some drawbacks such as requiring (i) a darkened room for testing, (ii) a 2 week training period for the animals, and (iii) low cost effectiveness compared to less expensive systems such as SFI. Despite these drawbacks, the CatWalk system has the clear advantage of being able to measure both dynamic and static gait parameter simultaneously. In the newer XT version of the CatWalk system, SFI can also be automatically calculated from a foot print analysis, and, thus, the system saves time as compared to making tedious SFI measurements. In this study, we found that subtle neurological alternations could be better detected by the new version of CatWalk XT system. In assessing neurological outcome, the Catwalk XT system appears to be able to provide a platform for assessing the benefits associated with early treatment of peripheral neurological injury.

## Conclusion

CatWalk XT system provided better temporal and imaging resolution than prior manual or computer aided neurobehavioral assessment tools. The new system proved capable of detecting subtle neurological alternation in a peripheral nerve injury model within as early as 7 days. These detections could not be achieved by SFI, angle of ankle with the CatWalk version 7. The Catwalk XT system can serve as a tool for quantitative and reliable assessment of treatment efficacy in peripheral nerve injury preclinical models.

## Abbreviations

SFI: Sciatic function index; HA: Hyaluronic acid; CMAP: Compound muscle action potential; NF: Neurofilament; S-100: S 100 proteins.

## Competing interests

The authors declare that they have no competing interests.

## Authors’ contribution

YJC carried out the animal study and drafted the manuscript. FCC participated in the study design and gait analysis. MLS participated in the study design and carried out the statistical analysis. HLS carried out the immunohistochemistry analysis. CJC participated in the study design and carried out the animal gait analysis. JS participated in the study design and help to draft the manuscript. HCP participated in the study design and coordination and helped to draft the manuscript. All authors read and approved the final manuscript.
